# Innovative Hydroponic Culture of *Alkanna tinctoria* (L.) Tausch: An Approach Towards Sustainable Extraction Process from Plant Roots

**DOI:** 10.3390/plants14192987

**Published:** 2025-09-26

**Authors:** Elodie Bossard, Annalisa Cartabia, Ismahen Lalaymia, Nikolaos Tsafantakis, Nektarios Aligiannis, Ioanna Chinou, Stéphane Declerck, Nikolas Fokialakis

**Affiliations:** 1 Division of Pharmacognosy and Natural Products Chemistry, Department of Pharmacy, School of Health Sciences, National and Kapodistrian University of Athens, Panepistimiopolis, 15771 Athens, Greecentsafantakis@pharm.uoa.gr (N.T.); aligiannis@pharm.uoa.gr (N.A.); ichinou@pharm.uoa.gr (I.C.); 2 Applied Microbiology, Mycology, Earth and Life Institute, Université Catholique de Louvain, 1348 Louvain-La-Neuve, Belgiumstephan.declerck@uclouvain.be (S.D.)

**Keywords:** *Alkanna tinctoria*, hydroponic system, nutrient film technique, natural deep eutectic solvent

## Abstract

*Alkanna tinctoria* (L.) Tausch is a valuable medicinal plant known for its root-derived hydroxynaphthoquinone enantiomers, alkannin/shikonin (A/S), which exhibit significant pharmaceutical and cosmeceutical potential. However, its limited natural distribution and overharvesting pose conservation challenges, necessitating sustainable cultivation and extraction strategies. The application of Natural Deep Eutectic Solvents (NaDESs) has garnered significant attention as sustainable alternatives to conventional solvents. However, their toxicity in living plant systems remains largely unexplored. This study presents the successful establishment of an ex situ hydroponic cultivation system using the nutrient film technique (NFT) to grow *A. tinctoria* under greenhouse conditions. The system promoted plant acclimatization, vigorous root development, and initial production of A/S derivatives. In parallel, the toxicity evaluation of a bio-based NaDES, LeG_5_20 (levulinic acid–glucose, 5:1, with 20% water), applied as a circulating medium, was assessed. Physiological stress responses of the plants to NaDES circulation were assessed through non-destructive measurements, including stomatal resistance, photosynthetic and transpiration rates, and sub-stomatal CO_2_ concentration. Short-term (24 min) exposure to NaDES showed no significant adverse effects, while longer exposures (4–8 h) induced marked stress symptoms and loss of leaf area. These findings demonstrate the feasibility of integrating green hydroponic systems with eco-friendly extraction solvents and provide a framework for further optimization of plant age, solvent exposure time, and system design to enable sustainable metabolite recovery without plant destruction.

## 1. Introduction

*Alkanna tinctoria* (L.) Tausch is a perennial herbaceous plant belonging to the Boraginaceae family, with a limited distribution in southern Europe [1]. In the external layer of its roots, it accumulates alkannin/shikonin (A/S), which are bioactive compounds characterized by a hydroxynaphthoquinone structure. Preparations made with Boraginaceae roots were commonly used in traditional medicine to treat wounds, burns, and ulcers [2]. In the past decades, several in vitro and in vivo studies have provided evidence of their medicinal properties. In fact, the hydroxynaphthoquinone enantiomers (HNQs) present anti-inflammatory [3], antimicrobial [4], and antitumor [5] activities, and especially wound healing properties [6]. They are currently applied as active ingredients in the marketed pharmaceutical formulation of Histoplastin Red, which is an effective wound healing agent. Therefore, HNQs are nowadays qualified as pharmaceutical and cosmeceutical agents [7].

Several efforts have been made to produce HNQs from plants grown under controlled conditions. Cell tissues and root cultures of *A. tinctoria* have been attempted [8,9], but the level of production remained insufficient, and even today, most of the pharmaceutical preparations worldwide containing A/S are still derived from roots of Boraginaceous species found in nature [10]. Moreover, the exploitation of *A. tinctoria* in the wild is putting this plant in danger of extinction, as already reported for other medicinal plants collected from their natural habitats (e.g., *Lithospermum erythrorhizon* in Japan, *Alkanna sieheana*, and *Alkanna orientalis* in Turkey) [11].

In nature, *A. tinctoria* reproduces through seeds enclosed in monospermic achenes characterized by a very low germination rate. Furthermore, the cultivation of Boraginaceae plants following conventional agriculture practices is not feasible due to several constraints (e.g., time needed for A/S production, high costs of harvesting, exposure to biotic/abiotic stresses). For these reasons, ex situ culture and preservation of this plant are carried out in a botanical garden in southern Europe. In recent studies, the wild plants collected were taxonomically identified and were given the International Plant Exchange Network (IPEN) accession number GR-1-BBGK-17,5975 for their long-term ex situ conservation (including sexual, asexual—vegetative, and in vitro propagation trials) at the premises of IPBGR, HAO-Demeter in Greece [12]. In addition, a hydroponic culture approach using *A. tinctoria* plants was introduced by Yankova et al. in 2020 [1]. Undoubtedly, the design of ex situ culture systems is a concern of the 21st century. In the context of an insatiable need for natural products, these cultivation system helps prevent extinction and overexploitation of several plant species [13]. As such, modern hydroponic systems are productive and promising methods, which offer new perspectives in the field of natural products [14]. In fact, they allow cocultures with beneficial microorganisms such as arbuscular mycorrhizal fungi (AMF), which have been demonstrated to stimulate the production of bioactive compounds [15]. Furthermore, these hydroponic systems are appropriate for the application of the Plant Milking Technology (Plant milking) (https://www.plantadvanced.com/home), which allows the direct trapping of active compounds in a non-destructive way using the same plant material multiple times [13].

Besides these promising cultivation techniques, eco-friendly solvents have also emerged. Over the last few years, Natural Deep Eutectic Solvents (NaDESs) have attracted increasing interest in the scientific community due to their low toxicity, biodegradability, and renewability properties [16,17]. They are defined as a mixture of two or three components capable of creating strong intermolecular interactions involving Van der Waals forces, electrostatic forces, and mostly hydrogen bonding. As a result, the eutectic mixture melting point is significantly lower than that of the precursor compounds [17]. NaDES are composed of natural products, such as amino acids, organic acids, sugars, and choline derivatives. Despite being mainly composed of hydrophilic primary metabolites, NaDESs are capable of solubilizing and extracting non-water-soluble and water-soluble molecules [18]. Interestingly, a tailor-made NaDES consisting of levulinic acid and glucose (LeG_5_20; 5:1 mol_HBA_:mol_HBD_; 20% water *w*/*w*) has previously been reported by Bossard et al. [19], demonstrating a high efficiency in extracting HNQs from *A. tinctoria* roots.

In the present study, an ex situ hydroponic cultivation system using a nutrient film technique (NFT) was developed for the growth of *A. tinctoria*. Furthermore, a proof of concept was challenged by combining our nutrient film technique (NFT) system with the use of LeG_5_20. Based on the fact that NaDESs reproduce the way secondary metabolites are solubilized and transported in plant cells [18], the conceptual aim of this study was to evaluate the direct effects of the customized NaDES LeG_5_20 on *A. tinctoria* plants within an ex situ hydroponic cultivation system. This work presents, for the first time, the preliminary results of NaDES toxicity assessment on *A. tinctoria*.

## 2. Results and Discussion

### 2.1. Circulatory Hydroponic System Using Nutrient Film Technique

**Figure 1:**
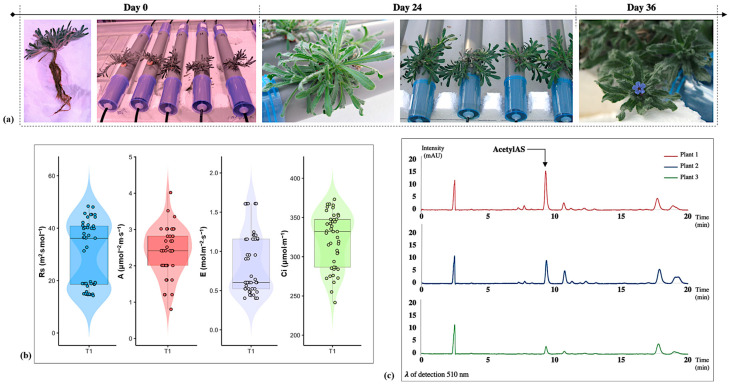
(**a**) Photographs of the circulatory hydroponic cultivation system of *Alkanna tinctoria* (L.) Tausch at different experiment times. (**b**) Leaf gas exchange parameters in *A. tinctoria* after four weeks of growing in the NFT system (*n* = 45; number of plants: 9; number of measurements/plants: 5). (**c**) HPLC-PDA chromatograms of the hydroxynaphthoquinone enantiomers contained in the roots of three different *A. tinctoria* plants after four weeks of growing in the NFT system (*λ* = 510 nm).

**Figure 2:**
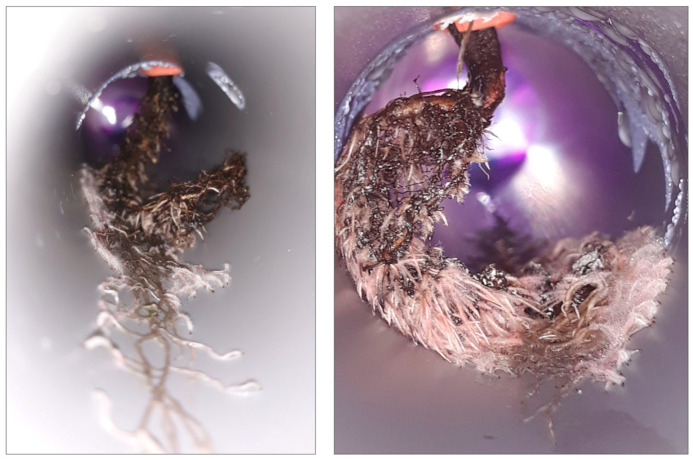
Photographs of the root biomass of *Alkanna tinctoria* after four weeks of growing in the NFT system (axial root and pinkish root branches).

As displayed in Figure 1a, the first trial of a hydroponic system for *A. tinctoria* using an NFT was successful. No mortality was reported; on the contrary, successful adaptation and significant growth until flowering were observed. Plant growth was noticed in view of the well-developed axial root and the numerous secondary root branches (Figure 2). Prior to the toxicity assessment, leaf gas exchange parameters were measured after four weeks of growing in the NFT system, to serve as reference values to check the homogeneity of the plant population (Figure 1b). Good homogeneity was measured for the photosynthetic rate (A). The distribution of values for the transpiration rate (E), leaf stomatal resistance to water vapor (Rs), and the sub-stomatal cavity CO_2_ concentration (Ci) was also homogeneous, mostly grouping together. A qualitative chemical analysis was then performed to evaluate the content of the hydroxynaphthoquinone enantiomers by HPLC-PDA using a specific wavelength of 510 nm (Figure 1c). As a result, the HNQ content was overall poor, which could be explained by the young age of the plants. Nevertheless, the main enantiomers were successfully identified as acetylshikonin-acetylalkannin (AcetylAS). To the best of our knowledge, this is the first report of both *A. tinctoria* cultivation and HNQ detection within an NFT system.

### 2.2. Assessment of NaDES Potential Toxicity on the Living Plant System

After four weeks of *A. tinctoria* acclimatization in the NFT system, the toxicity assessment began. Firstly, the NaDES stability was monitored under the greenhouse conditions: 24 °C/22 °C (day/night) and RH of 50%. Then, a volume of NaDES of 250 mL was set as the necessary volume to fill the pipes and the bottle sufficiently for continuous circulation. LeG_5_20 was circulated for twenty-four minutes, four hours, and eight hours, followed by twelve hours of Hoagland^mod^ nutritive solution circulation. Several parameters were measured during the experiment, such as Rs, A, E, and Ci, to monitor the effect of the NaDES solution on the plant.

**Figure 3:**
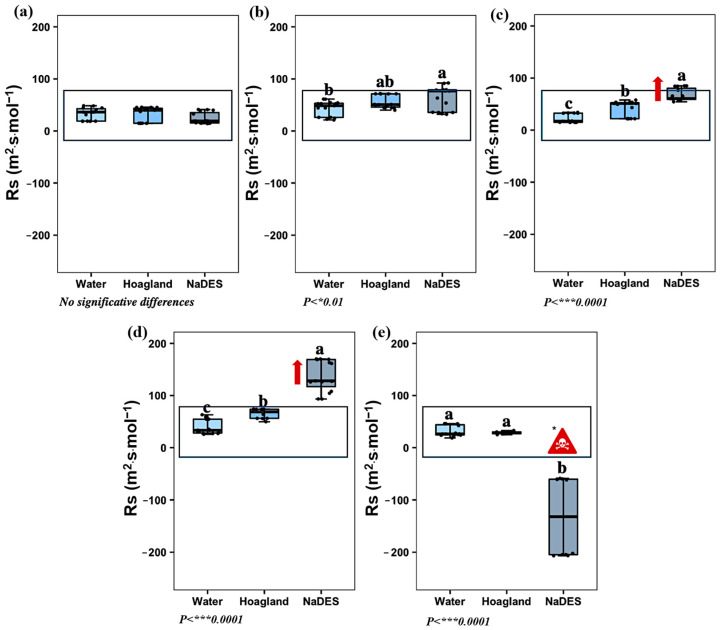
Effect of water, Hoagland^mod^ solution, and NaDES on the stomatal resistance to water vapor, Rs at (**a**) *T1: 4 acclimatization weeks. At T1, plant treatment groups (water, Hoagland^mod^, and NaDES) share the same circulating medium.; (**b**) T2: 24 min of water, Hoagland^mod^, and NaDES circulation; (**c**) T3: 4 h of water, Hoagland^mod^, and NaDES circulation; (**d**) T4: 8 h of water, Hoagland^mod^, and NaDES circulation; (**e**) T5: 12 h of Hoagland^mod^ solution. (Black dots: sample data points. Data are expressed on m^2^·s·mol^−1^, *n* = 15, HSD Tukey). Lowers letters ‘a’, ‘b’ and ‘c’ above the boxplots show the statistical difference. A significant deviation is highlighted by the red arrow. * An irreversible physiological damage is indicated by the symbol.

The dynamics of stomatal resistance (Rs) provide valuable insights into the plant’s stress response and its ability to regulate water loss and CO_2_ uptake. Under normal conditions, stomatal opening facilitates gas exchange, maintaining relatively low Rs values. In this study, plants treated with LeG_5_20 for 24 min maintained Rs levels similar to the control (around 53.7 m^2^·s·mol^−1^; Figure 3b), suggesting that this treatment did not immediately impair stomatal functioning. However, extended circulation of NaDES solutions induced progressive stomatal closure, as reflected in the substantial increase in Rs from 67.8 m^2^·s·mol^−1^ after four hours to 138.6 m^2^·s·mol^−1^ after eight hours, reflecting considerable stress in the plant (Figure 3c,d). After 12 h of Hoagland^mod^ solution circulation (Figure 3e), the stomatal function showed no signs of recovery (negative values of Rs). This may indicate irreversible physiological damage in the plants triggered by prolonged exposure to stress conditions.

**Figure 4:**
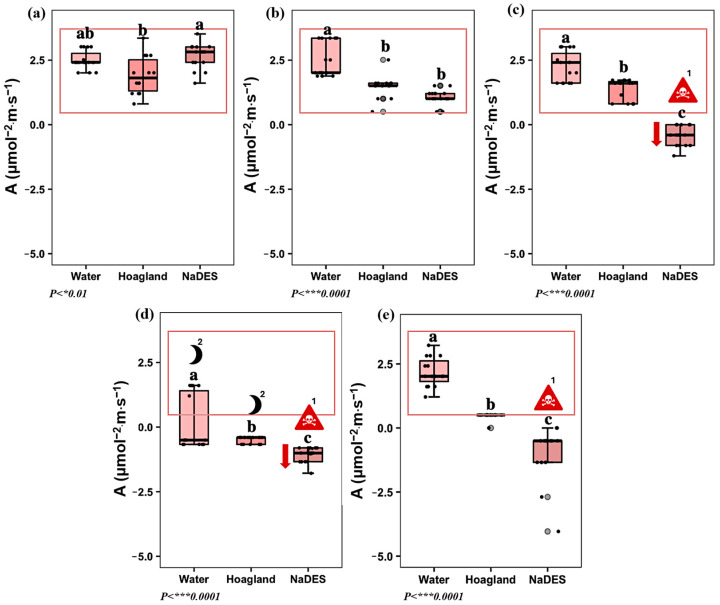
Effect of water, Hoagland^mod^ solution, and NaDES on the photosynthetic rate (A) at (**a**) *T1: 4 acclimatization weeks. At T1, plant treatment groups (water, Hoagland^mod^, and NaDES) share the same circulating medium.; (**b**) T2: 24 min of water, Hoagland^mod^, and NaDES circulation; (**c**) T3: 4 h of water, Hoagland^mod^, and NaDES circulation; (**d**) T4: 8 h of water, Hoagland^mod^, and NaDES circulation; (**e**) T5: 12 h of Hoagland^mod^ solution. (Black dots: sample data points. Grey dots: outside values. Data are expressed on μmol·m^−2^·s^−1^, *n* = 15, HSD Tukey). Lowers letters ‘a’, ‘b’ and ‘c’ above the boxplots show the statistical difference. A significant deviation is highlighted by the red arrow. ^1^ An irreversible physiological damage is indicated by the symbol. ^2^ Nighttime measurements.

The photosynthetic rate (A) closely reflected the plant’s diurnal rhythm, with reduced values during the night and enhanced activity during the day. Under stress conditions, however, this natural pattern is disrupted, with significant suppression of A even during the photoperiod. After 24 min of circulation, plants treated with LeG_5_20 exhibited photosynthetic rates comparable to those obtained with the Hoagland^mod^ solution (Figure 4b), suggesting that short-term exposure did not impair photosynthetic performance. After four hours of circulation, the A value of the plants treated with LeG_5_20 was significantly decreased from 1.07 μmol·m^−2^·s^−1^ to a negative value of −0.43 μmol·m^−2^·s^−1^ (Figure 4c). This clearly indicates a severe physiological decline in plants treated with NaDES at T3. After eight hours (T4), measurements were taken at night, a period where the photosynthetic rate naturally decreases in all plants (Figure 4d). Consequently, distinguishing between a stress response and the normal nighttime physiological state may be challenging at this stage. Nonetheless, the strong deviation from control values at earlier time points underscores the detrimental effect of long-term NaDES exposure. Following the recovery phase, photosynthetic activity in LeG_5_20-treated plants remained negative (–1.23 μmol·m^−2^·s^−1^) compared to the control plants, which regained daytime photosynthetic rates of 1.32 μmol·m^−2^·s^−1^ (Figure 4e). The transition from positive to negative values provides clear evidence of physiological collapse, underscoring the severe impact of prolonged NaDES exposure on the overall plant vitality.

**Figure 5:**
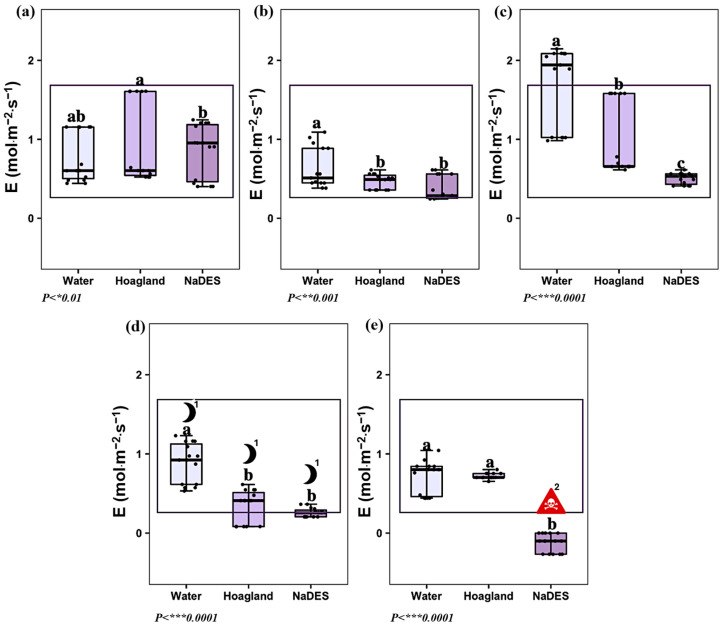
Effect of water, Hoagland^mod^ solution, and NaDES on the transpiration rate, E at (**a**) *T1: 4 acclimatization weeks. At T1, plant treatment groups (water, Hoagland^mod^, and NaDES) share the same circulating medium.; (**b**) T2: 24 min of water, Hoagland^mod^, and NaDES circulation; (**c**) T3: 4 h of water, Hoagland^mod^, and NaDES circulation; (**d**) T4: 8 h of water, Hoagland^mod^, and NaDES circulation; (**e**) T5: 12 h of Hoagland^mod^ solution. (Black dots: sample data points. Data are expressed on mol·m^−2^·s^−1^, *n* = 15, HSD Tukey). Lowers letters ‘a’, ‘b’ and ‘c’ above the boxplots show the statistical difference. ^1^ Nighttime measurements. ^2^ An irreversible physiological damage is indicated by the symbol.

The transpiration rate (E) exhibited a strong day–night rhythm, decreasing during the night and maintaining higher values during the day under non-stressed conditions. As with photosynthetic rate (A), stress strongly altered this natural cycle, leading to reduced transpiration even during the photoperiod. Throughout the experiment, the dynamics of E closely paralleled those of A. After four hours of circulation, plants treated with LeG_5_20 displayed significantly lower transpiration rates compared to the control group (Figure 5c). This decline is consistent with stomatal closure in response to NaDES-induced stress, which reduces both CO_2_ uptake and water vapor loss. After eight hours (T4), measurements coincided with the night period, when transpiration naturally decreases across all treatments (Figure 5d). At this stage, the additional effects of NaDES stress were not clearly distinguishable from the expected nighttime physiological decline. After the recovery phase, transpiration rates of LeG_5_20-treated plants dropped below zero (Figure 5e), in contrast to control plants that re-established positive daytime transpiration rates. These results indicate that stressed plants were unable to restore a normal water exchange process, highlighting an irreversible physiological impairment.

**Figure 6:**
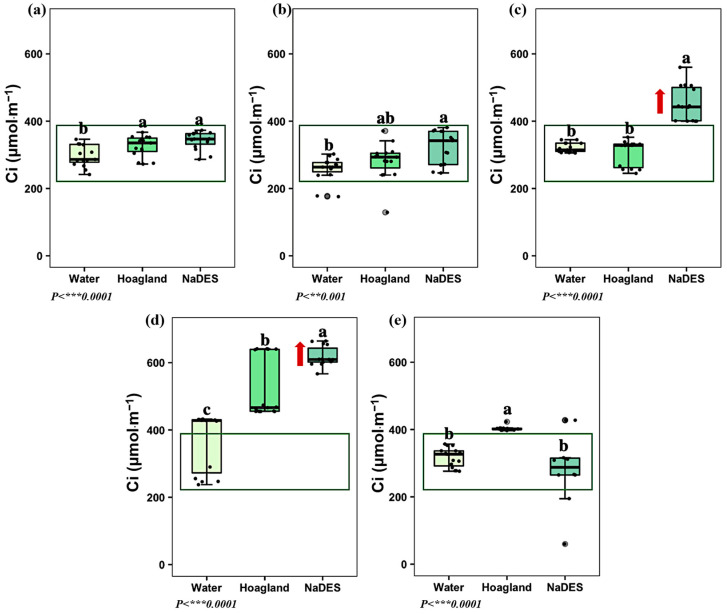
Effect of water, Hoagland^mod^ solution, and NaDES on the sub-stomatal cavity CO_2_ concentration, Ci at (**a**) *T1: 4 acclimatization weeks. At T1, plant treatment groups (water, Hoagland^mod^, and NaDES) share the same circulating medium; (**b**) T2: 24 min of water, Hoagland^mod^, and NaDES circulation; (**c**) T3: 4 h of water, Hoagland^mod^, and NaDES circulation; (**d**) T4: 8 h of water, Hoagland^mod^, and NaDES circulation; (**e**) T5: 12 h of Hoagland^mod^ solution. (Black dots: sample data points. Grey dots: outside values. Data are expressed on µmol·m^−1^, *n* = 15, HSD Tukey). Lowers letters ‘a’, ‘b’ and ‘c’ above the boxplots show the statistical difference. A significant deviation is highlighted by the red arrow.

Since plant cells need carbon dioxide (CO_2_) for photosynthesis, CO_2_ concentrations are another pivotal factor. Supposedly, when the CO_2_ concentrations inside the leaf start to decrease, the plant will open its stomata. As a result, more CO_2_ can enter, even under dry conditions when the stomata would ordinarily be closed. Furthermore, when the plant is under significant stress, the sub-stomatal cavity CO_2_ concentration (C_i_) increases, indicating that the CO_2_ cannot diffuse into the tissue anymore. In other words, the CO_2_ is not efficiently fixed due to impaired photosynthetic activity. After twenty-four minutes of treatment circulation, the C_i_ value of the plants treated with LeG_5_20 was equivalent to that of the control plants (Figure 6b). By contrast, significant increases in Ci were observed after four and eight hours of NaDES circulation (Figure 6c,d), reflecting a clear stress response and reduced efficiency of CO_2_ utilization within the photosynthetic machinery.

**Figure 7:**
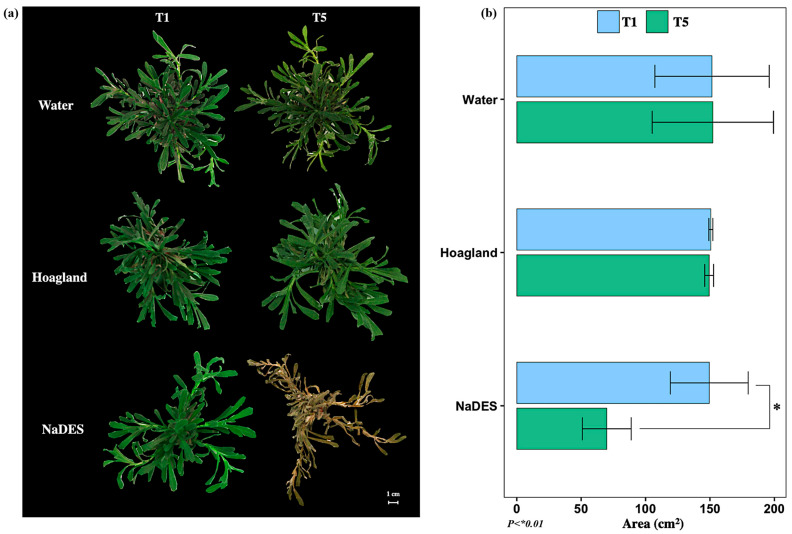
Morphology of *A. tinctoria* for image analysis. (**a**) Photographs of the leaf areas taken for the different treatments at T1 and T5, and (**b**) leaf area of plants under different treatments at T1: after 4 weeks of acclimatization and T5: 12 h of Hoagland solution.

Similar trends were confirmed through the aerial morphological analysis of *A. tinctoria* conducted before NaDES treatment (T1) and after the recovery phase with the Hoagland^mod^ solution (T5). As shown in Figure 7, plants supplied with water or Hoagland^mod^ solution displayed no noticeable morphological alterations, maintaining stable leaf development throughout the experiment. In contrast, NaDES treatment led to pronounced morphological damage, most clearly reflected in a significant reduction in leaf surface area. By the end of the experiment, plants exposed to NaDES had lost 46.8% of their leaf area (Figure 7b), underscoring the severity of the stress response. The marked reduction in leaf area indicates that the prolonged exposure to NaDES not only impaired physiological functions (as reflected by declines in A, E, and abnormal Ci and Rs dynamics) but also translated into visible structural damage. Loss of leaf area reduces the surface available for light capture and gas exchange, thereby exacerbating the decline in carbon assimilation and water regulation. Such morphological deterioration is often associated with leaf senescence, tissue desiccation, and stress-induced plant mortality.

To summarize all the non-destructive measurements performed, NaDES treatment caused significant stress in *A. tinctoria* plants after four hours and eight hours of circulation. However, optimistic results were obtained after twenty-four minutes of circulation. Indeed, no significant harmful effects were observed in the plants after a restricted time of LeG_5_20 circulation.

## 3. Materials and Methods

### 3.1. Chemicals

Compounds for the preparation of NaDESs, including D-(+)-glucose (G, 99%) and levulinic acid (Le, 98%), were purchased from Alfa Aesar (Kandel, Germany). Ultrapure water was received from the LaboStar apparatus (Evoqua LaboStar 4, Evoqua Water Technologies, Pittsburgh, PA, USA). *n*-Hexane (< 98%) was obtained from Merck (Merck KGaA, San Jose, CA, USA). HPLC grade acetonitrile (MeCN), HPLC grade methanol (MeOH), LC-MS grade formic acid, and LC-MS grade trifluoroacetic acid (99.5%; TFA) were purchased from Fisher Chemical (Thermo Fisher Scientific, Waltham, MA, USA).

### 3.2. Biological Material

*Alkanna tinctoria* rooted shoot explants (accession number 175975, propagation date: 5 March 2019) were provided by the Hellenic Agricultural Organization Demeter (IPBGR, HAO Demeter, Thermi—Thessalonikis, Greece) in April 2019. The plants were kept moist in nursery trays (56.5 × 36.5 × 4 cm) containing a peat moss (Terrahum, Klasmann-Deilmann, Gmbh, Germany) and perlite (Geoflor, Perlite Hellas S.A., Volos, Greece) (1:3, *w*/*w*) mixture. After three weeks, once the roots were adequately developed, the plants were transferred individually to 2 L pots containing a lava stone (0–3 mm, DCM, Groobendonck, Belgium), quartz 1–2 mm (N°1, Euroquartz, Liège, Belgium), and peat moss (DCM, Groobendonck, Belgium) (2:1:1, *w*/*w*/*w*) mixture. The substrate was previously autoclaved twice for 15 min at 121 °C. The plants were watered once a week with a Hoagland low P solution (i.e., 90% P-impoverished solution—Phosphorus = 6.245 mg L^−1^, and without ammonium nitrate, referred as Hoagland^mod^ throughout the text –see Cartabia et al. [15]) containing in mg L^−1^ deionized water: 826 mg L^−1^ Ca(NO_3_)_2_·4H_2_O; 357 mg L^−1^ KNO_3_; 45.1 mg L^−1^ KCl; 105.4 mg L^−1^ K_2_SO_4_; 50 mg L^−1^ KNO_3_; 27.4 mg L^−1^ KH_2_PO_4_; 120.4 mg L^−1^ MgSO_4_; 0.5 mg L^−1^ MnSO_4_·H_2_O; 1.4 mg L^−1^ H_3_BO_3_; 0.2 mg L^−1^ CuSO_4_·5H_2_O; 0.1 mg L^−1^ (NH_4_)_6_Mo7O_2_·4H_2_O; 0.6 mg L^−1^ ZnSO_4_·7H_2_O, and 19 mg L^−1^ Fe-EDTA. The pH of the solution was adjusted to 5.6 ± 0.2 before use. The plants were kept under greenhouse conditions set at 25 °C/17 °C (day/night), a relative humidity (RH) of 50%, a photoperiod of 16 h day^−1^, and a photosynthetic photon flux (PPF) of 120 mol·m^−2^·s^−1^.

### 3.3. Nutrient Film Technique System Set-Up

**Figure 8:**
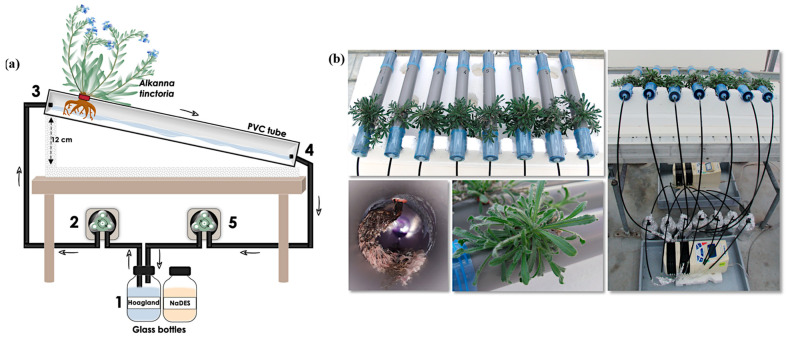
The NFT cultivation system applied for the toxicity assessment of NaDES on *A. tinctoria* plants. (**a**) Schematic representation of the system. The Hoagland solution and/or NaDES is circulated through the grey PVC tube supporting the *A. tinctoria* plant. The nutrient solution (or NaDES) in the glass bottle (1) is pumped using a peristaltic pump (2) to the upper part of the PVC tube (3) via black pipes. The solution percolates through the PVC tube (4) and is pumped back to the bottle using a second peristaltic pump (5). Grey arrows indicate the flow direction of the nutrient solution (or NaDES) in the tubing. (**b**) Pictures from the experimental setup conducted under greenhouse conditions at UCLouvain (system adapted from IJdo et al. [20].

**Figure 9:**
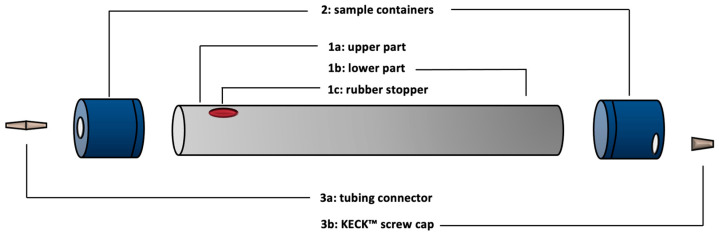
Schematic overview of the PVC grey tube. (1) Growth tube with (a) upper part (inlet); (b) lower part (outlet); (c) rubber stopper (cut and placed in a hole to allow plant insert). (2) Sample containers for closing the growth tube and on which a tubing connector (3a) and a KECKTM screw cap (3b) are glued to allow inlet and outlet tubing.

Ten-month-old *A. tinctoria* (16 in total) were gently removed from the 2 L pots, and their roots were cleaned with deionized water to eliminate substrate debris (January 2020). They were subsequently transferred to the NFT system (Figure 8) to begin the acclimatization process. This system, derived from the setup described by IJdo et al. [20], consisted of a grey PVC tube (500 mm length, 50 mm outer Ø, 40 mm inner Ø, 2 mm thickness, Martens) linked at both extremities to black pipes (4.6 mm, 3/16”, GARDENA, Louvain-la-Neuve, Belgium) connected to a 1 L glass bottle (SCHOTT DURAN, Mainz, Germany), covered with aluminum foil and filled with Hoagland^mod^ nutrient solution (or NaDES). This was circulated continuously via two multichannel peristaltic pumps (Gilson’s Minipuls Evolution, Villers-le-Bel, France): the first one pumped the solution from the bottle to the upper part of the growth tube (at 7 mL min^−1^) and the second one sucked the solution from the lower part (at 20 mL min^−1^) back to the bottle. To allow plant insertion, a hole (Ø 12 mm) was made in the upper part of the growth PVC tube, 150 mm away from the upper part (and therefore 350 mm from the lower part) (Figure 9). The growth PVC tube was inserted at the two extremities into two blue sample containers (100 mm height, 52 mm outer Ø, 50 mm inner Ø, 1 mm thickness, VWR, Leuven, Belgium). The left container was considered as the inlet cap (through which the liquid culture medium enters the growth tube) and the right one as the outlet cap (from which the liquid culture medium leaves the growth tube). A hole (Ø 6 mm) was made both at the center of the inlet to fix a tubing connector (47 mm length, 3–5 mm Ø, VWR, Leuven, Belgium) and at the edge of the bottom of the outlet to attach the Keck^TM^ screw cap cut (28 mm, 3–5 mm Ø, VWR, Belgium) (Figure 9).

A 20 °C slope was created using a flex foam support (Figure 8) to allow better flow of the solutions through the PVC tubes. The NFT systems were placed in a 16 m^2^ greenhouse set at the following conditions: 24 °C/22 °C (day/night), RH of 50%, photoperiod of 16 h light/8 darkness, and LED light (Lumigrow, Emeryville, CA, USA) with an intensity of about 220 μmol/m^2^/s.

The plants were acclimatized in the NFT systems for four weeks before proceeding with the NaDES toxicity assessment, using the Hoagland^mod^ solution diluted ×200, continuously flowing through the PVC tubes.

### 3.4. Preparation of NaDES

The NaDES, LeG_5_20, was prepared by mixing levulinic acid as the hydrogen bond acceptor (HBA), and D-(+)-glucose as the hydrogen bond donor (HBD), respecting the molar ratio 1:5 (mol_HBA_:mol_HBD_), with 20% of distilled water (*w*/*w*). The mixture was sonicated at 80 °C in a water bath (Elmasonic S100 H; Elma Hans Schmidbauer GmbH & Co. KG, Sin-gen, Germany), in a closed glass bottle, until a homogeneous transparent liquid was obtained.

### 3.5. HPLC-PDA Analysis of Hydroxynaphthoquinone Enantiomers

A microextraction of hydroxynaphthoquinone enantiomers was performed using *A. tinctoria* roots collected after four weeks of growth in the NFT system, immediately prior to the onset of the LeG_5_20 toxicity assessment. Dry powdered *A. tinctoria* roots (30 mg) of three different plants were subjected to ultrasound-assisted extraction using 1 mL of *n*-hexane at room temperature for 25 min. The obtained hydroxynaphthoquinone-enriched extracts were then evaporated under a steady stream of nitrogen, redissolved in 500 µL of HPLC-grade methanol, and filtrated using a 0.45 μm PVDF filter (RephiQuick Synringue Filter; RephiLe Bioscience, Ltd., Boston, MA, USA).

The qualitative analysis of hydroxynaphthoquinone enantiomers was performed using a hybrid system consisting of an ECOM ECP2000 Pump (ECOM spol. s r.o., Chrastany, Czech Republic) coupled with an ECB2000 gradient box with degasser (ECOM spol. s r.o., Chrastany, Czech Republic), a MISTRAL oven (Spark Holland BV., Emmen, The Netherlands) an ALLIAS autosampler (Spark Holland BV., Emmen, The Netherlands), and an ECDA Diode Array Detector (ECOM spol. s r.o., Chrastany, Czech Republic). The system was controlled using the DataApex Clarity™ Chromatography Software (version 9.1.1.13). The analysis was carried out on a UniverSil HS C8, 250 × 4.6 mm, 5 μm column at the flow rate of 1 mL/min using a mobile phase consisting of H_2_O, 0.1% TFA (A), and ACN (B) in an isocratic elution mode: 25% A and 75% B. The injection volume was set to 20 μL, and the detection wavelength was 510 nm. The main enantiomers, AcetylAS, were identified by comparing their retention time with those of known standards previously isolated and purified using *A. tinctoria* roots [19].

### 3.6. Toxicity Assessment of NaDES Extractive Solution

#### 3.6.1. Experimental Set-Up

**Figure 10:**
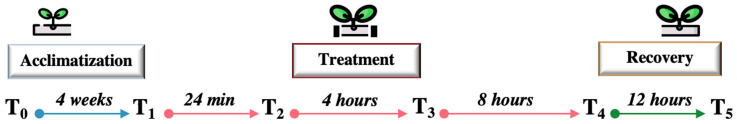
Experimental timing schema. Plants were exposed to NaDES (T1–T4) preceded by a four-week acclimatization period (T0–T1) and followed by 12 h of Hoagland solution circulation (T4–T5).

After four weeks (T1), in which the *A. tinctoria* plants were well acclimatized to the NFT systems, the toxicity experiment began. The plants were equally distributed on two tables following the set-up described above. The objective of the experiment was to evaluate the direct effects of the customized NaDES LeG_5_20 on *A. tinctoria* plants when used as a circulating medium at different exposure points within an ex situ hydroponic cultivation system. The experiment was performed on nine plants (three plants per treatment) flooded by different solutions: Hoagland^mod^, water, and NaDES. Each solution was circulated for twenty-four minutes (T2), four (T3), and eight (T4) hours continuously (Figure 10)**.** After each time of circulation, different plant stress parameters (i.e., leaf area, photosynthetic rate) were measured. Finally, the plant’s recovery was monitored for 12 h (T5) after the end of the experiment.

#### 3.6.2. Leaf Gas Exchange Parameters in *A. tinctoria* Growing in the NFT System

**Figure 11:**
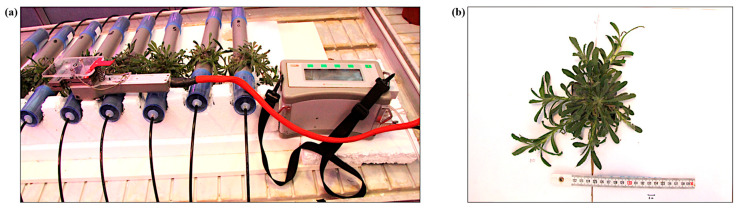
(**a**) Plant stress parameters measured by an infrared gas analyser (IRGA) at the UCLouvain greenhouse. (**b**) Example of setup for leaf area measurements.

Leaf stomatal resistance to water vapor (R_s_), photosynthetic rate (A), transpiration rate (E), and sub-stomatal cavity CO_2_ concentration (C_i_) were measured using an infrared gas analyzer (IRGA-LCi Photosynthesis system, ADC BioScientific Ltd., Hoddesdon, UK). An average of one measurement per minute over a 10 min period, recorded from a 4 cm^2^ leaf area, was considered for each plant in the analysis. The same measurement procedure was applied consistently across all time points (T_1_, T_2_, T_3_, T_4_, and T_5_) (Figure 11a).

#### 3.6.3. Arial Plant Area Measurement

In order to perform another non-destructive measurement on the plant’s growth/stress, the arial plant area was estimated through image analysis [21]. Pictures were taken with a Canon EOS 60D at a fixed distance from the plants, and the *A. tinctoria* rosette was placed under a white paper (to facilitate the image analysis) with a scale reference (Figure 11b). Image processing was conducted using the open-source image analysis software ImageJ (version 1.54k).

### 3.7. Statistical Analysis

All statistical graphs and data analysis (ANOVA and post-hoc Tukey test) were performed by RStudio version 1.2.5033 (2009–2019 RStudio, Inc., Boston, MA, USA) using the “ggplot2” [22] and “agricolae” [23] packages.

## 4. Conclusions

The development of an ex situ cultivation of *A. tinctoria* using a hydroponic NFT system was achieved with promising plant acclimatization and root growth. The preliminary results of the NaDES toxicity assessment led to an interesting reflection on the use of LeG_5_20 extractive solution on a healthy system and gave directions for further applications. Future experiments must consider a shorter circulation time and, potentially, older plant materials producing HNQs in high quantities. The main advantage of applying this system is the use of a green solution for capturing bioactive compounds from healthy plants. Knowing that NaDES are highly customizable solvents, this proof-of-concept opens the door for further study perspectives

## Data Availability

The original contributions presented in this study are included in the article. Further inquiries can be directed to the corresponding authors.
